# Subverting Host Defense from Within: Innate Immune Modulation by *Coxiella burnetii*

**DOI:** 10.3390/pathogens15040444

**Published:** 2026-04-20

**Authors:** Anna O. Busbee, Aryashree Arunima, James E. Samuel, Erin J. van Schaik

**Affiliations:** Department of Microbial Pathogenesis and Immunology, Naresh K. Vashisht College of Medicine, Texas A&M University, Bryan, TX 77807, USA; apinson@tamu.edu (A.O.B.); aarunima@tamu.edu (A.A.); jsamuel@tamu.edu (J.E.S.)

**Keywords:** *Coxiella burnetii*, innate immunity, macrophages, type IVB secretion system, immune evasion, pattern recognition receptors, inflammasome, interferon, effector-triggered immunity

## Abstract

*C. burnetii* (Cb) is an obligate intracellular bacterial pathogen that replicates within alveolar macrophages following aerosol infection. Unlike most intracellular bacteria, Cb establishes a lysosome-derived replicative niche (*Coxiella*-containing vacuole or CCV) through the action of its Type IVB secretion system (T4BSS). This system translocates a large repertoire of effector proteins into the host cytoplasm after phagosome acidification. These effectors interfere with diverse signaling pathways to co-opt host processes, such as vesicle trafficking, ubiquitylation, gene expression and lipid metabolism, promoting pathogen survival without triggering robust proinflammatory signaling or host cell death pathways. This effector-triggered immune silencing is particularly unique given the central role of macrophages as innate immune sentinels. In this review, we examine Cb T4BSS effectors that have been characterized as central determinants of innate immunity modulation. We discuss innate immune sensing pathways potentially engaged during infection, including Toll-like receptors, NOD-like receptors, RIG-I-like receptors, inflammasomes, and interferon signaling pathways, and highlight evidence indicating that these pathways are actively suppressed. Emphasis is placed on effector-mediated regulation of NF-κB signaling, type I interferon responses, and inflammasome activation. Finally, we address unresolved questions related to effector-triggered immunity, redundancy in immune suppression, and discrepancies between in vitro and in vivo infection models.

## 1. Introduction

*C. burnetii* (Cb) is a distinctive intracellular pathogen, most notably due to its ability to colonize macrophages without substantial induction of innate immune pathways [1]. Macrophages are one type of innate immune cell that are found throughout the body and at mucosal surfaces, including the lungs, where Cb infection begins. Under normal circumstances, macrophages recognize pathogens and differentiate into distinct phenotypes, upregulating a plethora of genes resulting in cytokine secretion [2]. It is significant that Cb can invade and replicate within these cells without eliciting such responses or triggering host cell death pathways, including apoptosis, pyroptosis, and necroptosis [3]. This phenotype is particularly striking given that macrophages function as innate immune sentinels responsible for pathogen detection, immune signaling, and microbial clearance. The limited activation of innate immunity during Cb infection can be partially attributed to modified bacterial pathogen-associated molecular patterns (PAMPs) such as lipopolysaccharide (LPS) that fail to efficiently engage canonical pattern recognition receptors (PRRs) and restrict host access to immunostimulatory ligands [4]. More importantly, Cb deploys a large repertoire of secreted effectors that actively suppress host signaling pathways and, even when partial activation occurs, inhibit downstream cell death programs [3,5,6]. In this review, we briefly introduce Cb and key components of the innate immune system, followed by an overview of Cb-host immune interactions and a focused discussion of effector proteins that modulate these pathways.

### 1.1. C. burnetii Infection in Humans and Animals

Cb is a Gram-negative intracellular pathogen and the etiological agent of Q fever in humans and coxiellosis in animals [6]. Human infection is primarily acquired through inhalation of aerosolized particles contaminated with Cb, typically originating from infected animal byproducts. There is no evidence that Q fever is transmitted from person to person, lending to its lower infection burden and association with people who work in close contact with ruminants [7]. The primary target cells during early infection are alveolar macrophages. Infection may be asymptomatic (up to 60% of cases) or present as acute Q fever, a self-limiting flu-like illness characterized by fever, retro-orbital headache, malaise, and fatigue [8]. In a subset of cases, acute disease can progress to atypical pneumonia or hepatitis. Following primary infection, approximately 1–5% of cases progress to chronic Q fever, most commonly manifesting as endocarditis [8]. Chronic Q fever is associated with a poorer prognosis and requires prolonged antibiotic therapy, often lasting 18–24 months [9]. Cb infects a wide range of animal species; however, domestic ruminants, including cattle, sheep, and goats, represent the primary reservoirs contributing to human infection. Animal infections are frequently subclinical, but when symptomatic, they are most commonly associated with reproductive disorders such as abortion, stillbirth, and infertility [10]. Shedding Cb in birthing products, feces, milk, and urine leads to environmental contamination. A meta-analysis of bacterial load in cow herd bulk milk tanks showed Cb prevalence amongst dairy farms to be 37% [11]. Owing to its efficient aerosol transmission, environmental persistence, and low infectious dose in humans, Cb is classified as a Category B Select Agent by the U.S. Centers for Disease Control and Prevention (CDC).

### 1.2. C. burnetii Intracellular Lifecycle

Cb exhibits a biphasic developmental cycle that begins with the environmentally stable and metabolically inactive small-cell variant (SCV), which is phagocytosed by alveolar macrophages (AM) following inhalation. In vitro studies have demonstrated that uptake is a passive process on the bacterial side, as live and dead Cb enter cells at similar levels; however, entry is dependent on the actin cytoskeleton [12]. The internalized SCV traffics through the default endolysosomal pathway, ultimately reaching the phagolysosome, where it matures into the metabolically active large-cell variant (LCV) to create its replicative niche, the *Coxiella*-containing vacuole (CCV) [13]. Establishment of this niche requires the Type IVB secretion system (T4BSS) [14,15,16]. Acidification of the CCV triggers metabolic activation and the SCV-to-LCV transition, coinciding with assembly and activation of the T4BSS [17]. Consistent with this, recent cryo-electron microscopy studies have demonstrated that SCVs lack T4BSS components in their membranes [18]. To date, approximately 100 T4BSS effector proteins have been identified and are secreted into the host cytoplasm, where they modulate diverse cellular pathways to support formation and maintenance of the replicative niche [5,6]. A comprehensive review by Ruart et al. details the discovery and functional characterization of these effectors [19]. The present review focuses specifically on effectors that target innate immune signaling pathways.

A defining feature of Cb infection is its capacity to replicate within primary murine bone marrow-derived macrophages (BMDM) for up to one week without inducing the typical inflammatory responses or host cell death [1,20,21]. At advanced stages of infection, CCVs can occupy the majority of the host cell cytoplasm—an outcome that is particularly striking given the macrophage’s role in pathogen detection and clearance [1,22]. The limited activation of innate immune pathways during infection is partly attributed to the bacterium’s poor immunostimulatory molecular patterns but is thought to rely predominantly on T4BSS effectors that actively suppress host signaling [6]. We postulate that before expression and secretion of T4BSS effectors, Cb PAMPs can trigger some activation through PAMP-triggered immunity (PTI), and that transition back to SCV coincides with cessation of T4BSS effector secretion, thereby allowing immune activation. This is supported by the observation that early on during infection there is an upregulation of cytokine expression [23]. We further hypothesize that Cb inhibits multiple effector-triggered immunity (ETI) mechanisms triggered by modifications of cellular homeostasis in a T4BSS dependent manner. In preparing this narrative review, we searched the literature using combinations of the key terms ‘*Coxiella burnetii*’ ‘type IV secretion system/effectors’, and ‘innate immunity’ in databases such as PubMed and Google Scholar. We then highlighted studies that provided particularly informative or illustrative mechanistic insights to best support the narrative focus of the review.

## 2. The Innate Immune System, PAMP-Triggered Immunity, and Effector-Triggered Immunity

**Figure 1:**
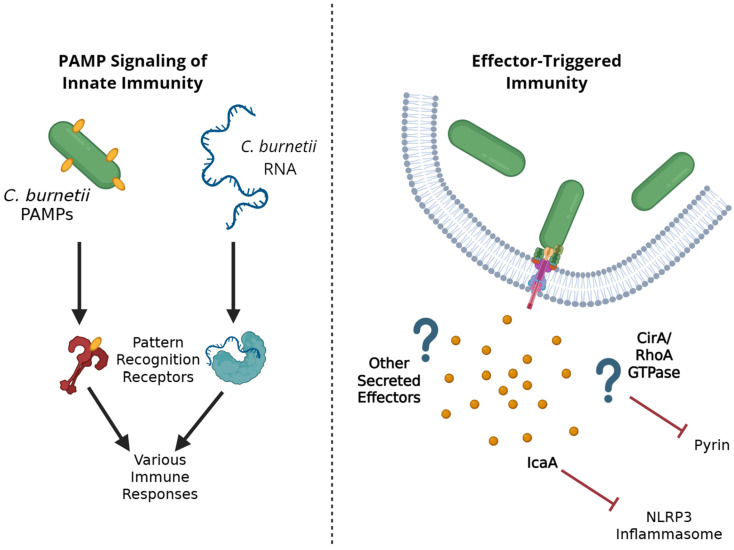
Summary of *Coxiella burnetii* interactions with host cell innate immune response. There are two main methods by which Cb can trigger host innate immunity. First, Cb PAMPs or genetic material stimulate PRRs, leading to typical host immune responses. Second, effectors themselves or the consequences of effector-mediated inhibition might stimulate host immune responses. Ultimately, Cb effectors suppress many of these notable innate immune responses, allowing for bacterial replication.

The innate immune system constitutes the first line of defense against infection and detects pathogens through dedicated germline-encoded receptors [24]. Unlike receptors of the adaptive immune system, innate immune receptors do not undergo genetic rearrangement, which underlies their designation as “innate.” This system is primed to sense both invading pathogens, including intracellular bacteria such as Cb, and cellular stress resulting from pathogen entry and intracellular replication. Pathogen detection is mediated by PRRs that recognize PAMPs [25], which are conserved microbial structures absent from mammalian cells. A classic example of a bacterial PAMP is LPS, an outer membrane component of Gram-negative bacteria and the primary mediator of endotoxic shock [26]. Engagement of PRRs by PAMPs initiates ligand–receptor interactions that activate downstream signaling cascades (Figure 1). The mechanisms underlying PTI, sometimes referred to as pathogen-triggered immunity, have been extensively characterized, in part because purified PAMPs enable direct interrogation of these pathways. PRRs involved in PTI include Toll-like receptors (TLRs), C-type lectin receptors (CLRs), retinoic acid-inducible gene I-like receptors (RLRs), and nucleotide-binding domain, leucine-rich repeat-containing receptors (NLRs), including pyrin and Z-DNA-binding protein 1 (ZBP1) [27,28]. In addition to PTI, host cells employ ETI, a surveillance strategy that detects pathogen-mediated perturbations of host cellular processes rather than non-self molecular structures [29]. This was originally discovered in plants where NLRs directly or indirectly identify pathogenic effectors, resulting in a conformational change and a signaling cascade leading to the hypersensitive response (HR) [30]. Direct interaction in plants occurs when a resistance protein directly recognizes a pathogenic protein to initiate an immune response. The indirect mechanism occurs when a guard protein recognizes alterations in cellular homeostasis, which is the mechanism we will discuss. ETI relies on identification of pathogen manipulation of host cells, usually accomplished through the secretion of effectors into the cytoplasm, which results in the ‘violation of cytoplasmic sanctity’ (Figure 1) [31]. ETI has also been described as detecting homeostasis-altering molecular processes (HAMPs). Another ETI mechanism is the release of danger-associated molecular patterns (DAMPs) that occurs through ‘violation of cytoplasmic sanctity’ caused by pathogenic bacteria instead of sterile inflammation. This is a more discriminative threat platform because commensal bacteria also encode PAMPs, but only pathogenic bacteria and their effectors gain access to the cytoplasm. Given that Cb is a bacterium possessing both PAMPs and cell pathway-modulating effectors, we would expect it to induce both PTI and ETI simultaneously. It is known that Cb triggers disruptions of cellular homeostasis through the secretion of its ~100 effectors into the cytoplasm, which manipulates numerous cellular pathways [32,33,34]. Although no specific mechanisms have been identified, it likely triggers numerous ETI responses. ETI mechanisms often occur through the activation of inflammasomes and cytosolic receptors like NOD1/2 and cGAS-STING. Additionally, inhibition of cellular processes like protein translation induces cytokine production and can be said to produce an ETI response [35,36,37]. Below, we describe the innate immune receptors identified as capable of recognizing Cb and its PAMPs or as participating in potential ETI mechanisms.

### 2.1. Toll-like Receptors

TLRs are membrane-bound pattern recognition receptors that share a conserved domain architecture consisting of an N-terminal ectodomain composed of leucine-rich repeats, a single transmembrane domain, and a cytosolic Toll/interleukin-1 receptor (TIR) domain [38]. Different organisms encode distinct repertoires of TLRs; relevant to this review, mice express 12 TLRs (TLR1–TLR9 and TLR11–TLR13), whereas humans express 10 TLRs (TLR1–TLR10) [39]. These receptors are localized either at the plasma membrane or within endosomal compartments. Cell-surface TLRs include TLR2/1, TLR2, TLR4, TLR5, and TLR2/6, while endosomal TLRs include TLR3, TLR4, TLR7, TLR8, TLR9, and TLR13. Engagement of TLRs by their cognate pathogen-associated molecular patterns induces receptor dimerization and initiates downstream signaling cascades. Dimerization promotes the assembly of supramolecular organizing centers (SMOCs) at the cytosolic TIR domains, which function as platforms for signal transduction. Two principal SMOCs have been defined: the Myddosome, assembled downstream of TLR2/1, TLR2, TLR4, TLR5, TLR2/6, TLR7, and TLR9, and the Triffosome, assembled downstream of TLR3 and TLR4 [40]. Although these complexes differ in composition, both activate signaling pathways that culminate in transcriptional responses leading to the production of pro-inflammatory cytokines, type I interferons, chemokines, and antimicrobial peptides. These transcriptional programs are primarily mediated by the transcription factors (TF) nuclear factor κB (NF-κB), interferon regulatory factor 3 (IRF3), and activator protein-1 (AP-1). Cb produces PAMPs that should theoretically be able to signal through all the TLRs except TLR5 because Cb is non-motile and does not encode flagellar genes. However, the capacity for Cb-specific PAMPs to signal through all the other TLRs is an incomplete story, as will be discussed further in Section 5.1 below.

### 2.2. Nucleotide-Binding Leucine Rich Repeat Receptors (NLRs)

NLRs, a group of cytoplasmic PRRs, share a conserved structural organization consisting of three core domains: an N-terminal variable effector domain, a central nucleotide-binding domain, and a C-terminal leucine-rich repeat domain [41]. The N-terminal domain mediates downstream signaling by facilitating protein–protein interactions that promote assembly of inflammasomes. Inflammasomes are supramolecular cytoplasmic complexes that drive inflammatory responses and programmed cell death. Each inflammasome is defined by its sensor PRR, including NOD1, NOD2, AIM2, NLRP1, NLRP3, NLRP6, NLRP9b, pyrin, and caspase-4/5/11 [14]. Upon activation, these sensors recruit adaptor proteins containing caspase activation and recruitment domains (CARDs) through pyrin domain (PYD) interactions, linking them to inflammatory caspases via CARD–CARD interactions. An exception is NLRC4, which can directly recruit caspase-1 through its CARD domain. Recruitment of caspase-1 results in autocatalytic processing and generation of the active enzyme, which subsequently cleaves pro-interleukin-1β (pro-IL-1β), pro-IL-18, and gasdermin D (GSDMD), culminating in cytokine release and pyroptotic cell death. At least in vitro, inflammasome activation requires two signals: a priming signal (signal 1) that induces transcriptional upregulation of inflammasome components such as NLRP3 and pro-IL-1β, followed by an activation signal (signal 2) that promotes inflammasome assembly. Cb possesses PAMPs capable of triggering signal 1 through TLR engagement, and infection has been shown to increase IL-1β expression [23]. The bacterium also encodes PAMPs that could potentially activate NOD1, NOD2, AIM2, and caspase-4/5/11; however, access to these ligands is limited by replication within the CCV. Alternative activation through caspase-11 is designated as the non-canonical inflammasome activation pathway, typically triggered by Gram-negative bacteria LPS or toxins in the cytosol, although this perspective is constantly evolving [42]. Notably, other vacuolar pathogens, such as *Salmonella enterica* serovar Typhimurium, release PAMPs into the cytosol through Type III secretion systems (T3SS), including flagellin [43,44].

NAIP (neuronal apoptosis inhibitory protein) family members are NOD-like receptors that combine anti-apoptotic baculoviral IAP repeat (BIR) domains with canonical NLR motifs, enabling them to detect specific bacterial ligands and regulate inflammasome activation. NAIP proteins directly sense components of the T3SS and flagellin proteins, from pathogens such as *S. Typhimurium*, enterohemorrhagic *Escherichia coli*, *Shigella flexneri*, and *Burkholderia* species. Upon ligand engagement, human NAIP (hNAIP/NAIP1) and murine NAIP2/5 assemble into large oligomeric complexes with NLRC4 in the presence of their cognate bacterial ligands, thereby supporting ligand-specific reconstitution of the NLRC4 inflammasome. These findings suggest that analogous surveillance mechanisms may exist for other bacterial secretion systems, including T4BSS, although such mechanisms have yet to be identified [45]. In addition, recent studies have revealed heterogeneity in CCVs, including a transiently leaky phenotype during in vitro infection, which may permit the release of PAMPs into the cytoplasm [46].

### 2.3. Retinoic Acid-Inducible Gene I (RIG-I)-Like Receptors (RLRs)

RLRs are cytoplasmic pattern recognition receptors classically associated with antiviral immunity and are characterized by a central RNA helicase domain and a C-terminal domain (CTD). RLR signaling can exert either protective or detrimental effects during bacterial infection, depending on the pathogen and host context [47]. Recent studies have demonstrated that type I interferons restrict Cb growth and that Cb encodes effector proteins capable of inhibiting RIG-I signaling [48]. The RLR family includes RIG-I, melanoma differentiation-associated protein 5 (MDA5), and laboratory of genetics and physiology 2 (LGP2). RIG-I and MDA5 possess N-terminal caspase activation and recruitment domains (CARDs) that mediate downstream signaling, whereas LGP2 lacks CARDs and is thought to modulate the activity of the other RLRs. These receptors detect non-self RNA species in the cytoplasm, and ligand binding induces conformational changes that expose the CARDs of RIG-I and MDA5, enabling interaction with mitochondrial antiviral signaling protein (MAVS) and initiation of downstream signaling cascades. Although RLRs are best known for sensing viral RNA, accumulating evidence indicates that bacterial RNA and regulatory host RNAs can also activate these pathways [49]. The existence of a Cb effector (EmcB) that inhibits RIG-I signaling suggests the presence of a PAMP capable of engaging RLRs during infection [48]. While this may initially appear unlikely, Cb expresses small RNAs that could form dsRNA structures capable of RLR activation [50]. The mechanism by which such RNA species gain access to the cytoplasm remains unclear; however, potential routes include the release of bacterial RNA via outer membrane vesicles (OMVs) during infection [51] or transient permeability of the CCVs at specific stages of intracellular replication [46]. Thus, both the identity of the RLR-activating PAMP and the mechanism by which it reaches the host cytoplasm remain important unresolved questions.

### 2.4. cGAS-STING Pathway

While direct evidence for the presence of cytosolic Cb DNA during infection is currently lacking, the inhibition of RIG-I signaling suggests that Cb-derived dsRNA gains access to the host cytoplasm, allowing for the possibility that Cb simply releases its own DNA. Cytoplasmic DNA is sensed by the cGAS-STING pathway. Upon binding to DNA, cGAS catalyzes the production of the second messenger 2′3′-cyclic GMP–AMP (cGAMP), which in turn activates STING [52]. Unlike TLR9, which specifically recognizes unmethylated CpG motifs within single-stranded DNA, cGAS responds to the presence of any DNA in the cytoplasm, regardless of its origin. DNA binding to cGAS promotes STING dimerization and relocalization, potentiating recruitment of TANK-binding kinase 1 (TBK1). This signaling cascade culminates in the induction of pro-inflammatory cytokines and type I interferon responses. Recently, it was determined that Cb activates cGAS-STING at late timepoints during infection [21]. This suggests two possibilities for the Cb interaction with cGAS-STING machinery. First, it is possible that Cb effectors inhibit cGAS-STING during early infection and then cease to be secreted by the bacteria. Second, Cb DNA might not be secreted until later time points during infection. We hypothesize that Cb DNA can gain access to the cytoplasm either through the T4BSS, through the lysosomal leakage phenotype, or OMV release [46,51]. However, there are effectors that inhibit this process early during infection, highlighting a potential future role for spatial-temporal studies to better understand the intricate timing associated with Cb infection. The transition to SCV later during infection, however, would coincide with the cessation of effector secretion (as SCVs do not express T4BSS). This was supported by data indicating that *dotA* expression was reduced at later time points in WT BMDMs and that more Cb DNA was found in the cytoplasm at late time points compared to STING mutant BMDMs [18].

## 3. Innate Immune Pathways Detrimental to *Coxiella burnetii*

Why is inhibition of these pathways so important for Cb? These answers were determined before the identification of the T4BSS as essential for virulence and the characterization of effectors that modulate innate immunity. Although IFNγ is considered an innate immune pathway, the major producers of IFNγ are Cb antigen specific CD4+ T-cells which are only produced after an adaptive immune response [53,54]. Therefore, although these pathways are described as being innate immune, it is with the caveat that IFNγ is produced after an adaptive immune response. The major detrimental consequence of innate immune activation is secretion of cytokines and chemokines that activate infected macrophages to kill intracellular Cb. Historically, it has been known for decades that the addition of TNFα and especially IFNγ inhibit Cb growth in vitro [55,56]. Type I interferons are stimulated by several PPRs, and the major consequence of NF-κB activation is TNFα. Macrophages both produce and respond to TNFα through several pathways, complicating our understanding of the balance between production and autocrine signaling.

### 3.1. Type I Interferon

Type I interferons comprise a family of cytokines that signal through the type I interferon receptor, a heterodimer composed of IFNAR1 and IFNAR2. This family includes IFNω, IFNκ, IFNε, IFNβ, and 13 IFNα subtypes [57]. Ligand binding to the receptor rapidly activates the Janus kinase–signal transducer and activator of transcription (JAK–STAT) signaling cascade, culminating in the formation of a phosphorylated STAT1–STAT2 heterodimer that associates with interferon IRF9 to form the ISGF3 transcriptional complex. JAK1 mediates sequential phosphorylation of STAT1 at Tyr701 and Ser727. The ISGF3 complex translocates to the nucleus, where it induces the expression of interferon-stimulated genes (ISGs), including *Ifit1*, *Mx1*, *Viperin*, and *Irf1* [58]. Notably, interferons themselves are ISGs, creating a positive feedback loop that amplifies IFNα production and enhances cellular responsiveness to IFNγ. Although historically associated with antiviral immunity, type I interferons are now recognized as important modulators of antibacterial responses, including the response to Cb [21,48,59]. Additionally, vaccination with formalin-fixed Cb causes a general antiviral state indicating that it expresses PAMPs that can induce type I interferons [60].

Cb infection induces both type I and type II interferon signaling, and prior infection confers protection against subsequent challenge with interferon-sensitive viruses. Additional studies using IFNAR^−/−^ mice infected with NMI demonstrated that they lost less body weight than WT mice and that treatment of WT mice with intra-tracheal rIFN-α prevented dissemination to the spleens [59]. The addition of type I interferon, specifically IFNβ, to Cb-infected cells reduces the rate of bacterial replication [21,48]. The recent identification of two T4BSS effectors that modulate interferon signaling indicates that inhibition is a priority for Cb [48].

### 3.2. Type II Interferon

Type II interferon consists of a single cytokine, IFNγ. Although its downstream signaling shares similarities with type I interferon pathways, several key distinctions exist. The type II interferon receptor is a heterodimer composed of IFNGR1 and IFNGR2 subunits. Both JAK1 and JAK2 are associated with the receptor complex, with JAK1 mediating downstream phosphorylation of STAT1. STAT1 is phosphorylated at Tyr701 and Ser727, analogous to type I interferon signaling. The phosphorylated STAT1 homodimer subsequently translocates to the nucleus, where it binds interferon-γ-activated sequence (GAS) elements to induce transcription of interferon-stimulated genes (ISGs).

A prominent IFNγ-induced effector is inducible nitric oxide synthase (iNOS), which catalyzes the production of reactive nitrogen species with antimicrobial activity. IFNγ was previously shown to suppress Cb growth in mouse fibroblasts [55]. Consistent with this role, Andoh et al. demonstrated that IFNγ^−/−^ mice exhibit markedly increased susceptibility to Cb infection, accompanied by accelerated disease progression compared to wild-type animals. Furthermore, IFNγ^−/−^ mice were susceptible to Cb-induced febrile responses following infection with the avirulent Nine Mile II (NMII) strain, underscoring the critical role of IFNγ-mediated immunity in controlling infection in mice [53]. It should be noted that human iNOS is not significantly expressed due to epigenetic silencing; therefore, the true role of IFNγ in human models regarding iNOS specifically has yet to be determined [61].

### 3.3. NF-κB

Another critical signaling pathway central to both innate and adaptive immunity is mediated by the NF-κB family of TFs. In mammals, this family comprises RelA (p65), RelB, cRel, p50, and p52, which form various homo- and heterodimers that regulate transcription by binding specific DNA elements. Tight regulation of NF-κB signaling is essential and coordinated by the NF-κB essential modulator (NEMO), the regulatory subunit of the IκB kinase (IKK) complex [62]. NF-κB controls the expression of a broad range of genes involved in immune responses and cell survival, including anti-apoptotic proteins (e.g., Bcl-2), cytokines and chemokines (TNFα, IL-1α/β, IL-2, IL-6, IL-17), and cell cycle regulators such as cyclins D1 and D3. Notably, Cb has recently been shown to inhibit IL-17 signaling [63], a pathway closely linked to NF-κB activation. NF-κB signaling occurs through two main pathways: the canonical and non-canonical pathways. The canonical pathway is rapid and transient and is mostly associated with responses to infection. It is typically initiated by TLR engagement and culminates in the phosphorylation and nuclear translocation of the p50/RelA heterodimer, which functions as a transcription factor. Activation of NF- κB leads to the production of TNFα, which is once again detrimental to intracellular Cb growth.

Several Cb effectors have been implicated in the modulation of NF-κB signaling [64]. Cb infection preferentially engages the canonical NF-κB pathway, particularly involving RelA. Increased phosphorylation of RelA has been observed in Cb-infected THP-1 cells compared to uninfected controls, and pharmacological inhibition of NF-κB signaling modestly reduces intracellular bacterial replication. Importantly, this modulation is dependent on a functional T4BSS [65]. Collectively, these findings suggest that Cb maintains a finely tuned balance between NF-κB activation and inhibition that ultimately favors bacterial replication. One such effector, NopA, interacts with the small GTPase Ran and increases intracellular levels of Ran-GTP. Given Ran’s central role in nucleocytoplasmic transport, this interaction was examined in the context of NF-κB signaling, revealing that Cb infection impairs the nuclear translocation of RelA [64]. This mechanism provides a means by which Cb selectively dampens NF-κB-dependent transcription while avoiding overt immune activation. Table 1 below summarizes all the above-discussed innate immune mechanisms in the context of Cb infection.

## 4. Potential *Coxiella*-Stimulated ETI

Why have we not identified any ETI-induced mechanisms by Cb? The most succinct answer is that Cb in vitro infections do not result in cellular death, which is the usual outcome after triggering ETI. However, Cb secretes ~100 effectors into the cytoplasm of infected alveolar macrophages, which modify a plethora of critical signaling pathways without significantly alerting any innate immune pathways to the disruption of cellular defenses. This “violation of cytoplasmic sanctity” does not induce massive innate immune responses, indicating that a highly sophisticated host–pathogen tug-of-war is occurring. Cb seemingly turns off most host innate immune pathways before a response can be mustered. Although there are no published accounts of effectors that trigger ETI responses, we hypothesize that numerous effectors must exist that prevent these responses. It is out of the scope of this review to provide a thorough discussion of the effectors that prevent apoptosis; however, we would refer interested readers to Ruart et al. to understand how cellular death may be avoided after triggering ETI [19]. Here we will describe observations that should lead to an ETI response but do not, and it is our hypothesis that Cb encodes effectors that stop all ETI responses downstream of these recognized patterns of pathogenesis [66].

### 4.1. RhoA ETI Mechanisms

One of the most studied ETI mechanisms is the inactivation of RhoA by bacterial pathogens. This interaction triggers the pyrin inflammasome because, under conditions of normal cellular homeostasis, RhoA phosphorylates and inactivates pyrin. For example, TcdB from *Clostridium difficile* is endocytosed into host cells where it monoglucosylates RhoA thereby inactivating it. It was determined early on that RhoA was recruited to the CCV in a T4BSS-dependent manner [67]. Cb encodes an effector CirA that is essential for replication and toxic when ectopically expressed in yeast and mammalian cells [68]. Interestingly, it was determined that overexpression of RhoA in yeast suppressed this toxicity, suggesting that CirA inhibits RhoA. Furthermore, CirA acts as a GTPase for RhoA, which, if left unchecked, would cause significant inactivation of RhoA, ultimately leading to the activation of pyrin. Therefore, although it has not been investigated, it is likely that ectopic expression of CirA causes cell death through an ETI, most likely through the pyrin inflammasome. Additionally, Cb infection does not cause toxicity to the host cell, indicating that the activity of CirA is tightly regulated to either not cause activation of pyrin, or, in the wake of activation, inhibit the downstream inflammasome through the action of some unidentified effector [1].

### 4.2. Potential Cb ETI Mechanisms

It is likely that all the identified toxic effectors trigger ETI mechanisms and lead to cellular death. This points to spatial-temporal regulation of effectors as the key to the maintenance of the replicative niche, as any dysregulation of this process would lead to cellular death through ETI mechanisms. This also makes it extremely difficult to study the mechanisms that activate ETI or PTI. Furthermore, distinguishing between PTI and ETI activation is also extremely difficult because Cb does not cause any major activation [1]. These pathways can also overlap, such as in the case of the Cb effector IcaA. This effector, which will be discussed in further detail below, inhibits the non-canonical inflammasome activation initiated by caspase-11 recognition of LPS [69]. Activation of caspase-11 causes pyroptosis via secondary activation of NLRP3 through K^+^ efflux, hence the nomenclature non-canonical [70]. Therefore, a PTI mechanism causes secondary activation of an ETI mechanism. Even though IcaA inhibits the non-canonical inflammasome, the mechanism was only discovered during coinfection with *L. pneumophila* [69]. This begs the question: how do we study mechanisms of ETI inhibition? How will we determine what triggers these pathways in the first place? There is very little difference in the activation of innate immune signaling pathways in the first 24 h between wild-type and T4BSS deficient mutants, pointing to the conclusion that Cb does not inherently stimulate many innate immune pathways [1]. Remarkably, killed Cb cannot initiate these signaling pathways, even though purified Cb LPS does trigger innate immunity, suggesting that a combination of factors is involved, including the segregation of PAMPs from the pathway within the vacuole. However, once effectors gain access to the cytoplasm, a plethora of cellular modulations should stimulate an ETI response. The fact that no activation of inflammasomes is observed raises the possibility of numerous unidentified effectors that inhibit these responses.

## 5. *Coxiella burnetii* Experimental Challenges

Interpretation of Cb interactions with the innate immune system is complicated by several experimental and biological factors. Notably, important differences exist between virulent Select Agent strains and the avirulent, rough BSL-2 NMII isolate. Within the Select Agent strains, there also exists high variability in the strength of the host response, possibly due to differences in functional secreted effectors [5]. In addition, studies across laboratories vary widely in multiplicity of infection, experimental models, and whether infections are conducted in vitro or in vivo, with many reports representing isolated observations rather than standardized comparisons. Together, these variables make it difficult to assess the physiological relevance of individual findings and highlight the need for more systematic investigation. This section will highlight the challenges and nuances intrinsic to the study of this unique stealth pathogen.

### 5.1. Coxiella burnetii LPS

One of the most perplexing aspects of Cb biology and its interaction with the innate immune system concerns the LPS molecule. The Lipid A structure of LPS from the Select Agent isolates and rough NMII are identical and interact with TLR4 in the same manner [4]. This Lipid A is an atypical structure and does not cause the in vitro inflammatory response induced by classic *E. coli* LPS. More specifically, Cb Lipid A antagonizes the ability of *E. coli* LPS to signal through TLR4 [4]. This is because Cb LPS is modified to limit detection by TLR4 through acylation patterns. TLR4 and MD2 interact with the Lipid A structure of LPS, and structural studies have demonstrated that five of the six acyl chains present in hexa-acylated Lipid A interact with a hydrophobic pocket of the TLR4-MD2 heterodimer and the sixth acyl chain interacts with a different TLR4 molecule [71]. The Lipid A from Cb only contains four acyl chains, and like other Lipid A structures with less than six acyl chains, it should have minimal ability to crosslink TLR4-MD2 heterodimers, resulting in less activation [72].

In TLR4^−/−^ mice, Cb uptake, but not intracellular replication, was suppressed, indicating that interaction with TLR4 is critical for initial bacterial entry into the macrophage [73]. This same study displayed filamentous actin reorganization induced by Cb LPS that was not present in TLR4^−/−^ mouse macrophages. Paradoxically, NMI LPS does not allow activation of p38α-MAPK downstream of TLR4 activation via inhibition of TLR2 and TLR4 association [74]. Cb LPS might antagonistically interact with TLR4, blocking its TLR2 association but inducing actin reorganization to permit bacterial uptake. Interestingly, it was recently demonstrated that TLR2/TL4 heterodimers are involved in the recognition of atypical LPS structures, which suggests that Cb LPS may signal through this pathway, as both TLR2 and TLR4 knockout mice were more susceptible to infection compared with WT mice [75,76]. TLR2 has also been implicated in host recognition of Cb, as bacterial replication is enhanced in TLR2^−/−^ cells and disease severity is modestly increased in TLR2^−/−^ mice. Whether TLR2, TLR4, or heterodimeric TLR signaling contributes to this response remains unresolved.

Murine models do not fully recapitulate human Q fever, limiting the interpretation of these results. Guinea pigs more accurately model the consolidated pneumonia observed in humans, but the lack of available immunological reagents restricts mechanistic studies. Consequently, significant questions remain regarding the pathways governing innate immune recognition and control of Cb infection. The O-antigen, which is absent in NMII, contains unique sugars not found in other LPS structures, including virenose (Vir) and dihydrohydroxystreptose (Strep), which serve as biomarkers of Cb [72]. A significant component of the protective immunity conferred by the formalin-inactivated vaccine licensed in Australia (Q-Vax) is mediated by antibodies directed against the O-antigen, which is present only in smooth variants. Consistent with this, formalin-fixed rough variants fail to confer the same level of protection [64,77].

These observations suggest that, despite its atypical structure and poor TLR4 agonism, Cb LPS must engage immune pathways that contribute to protective immunity; otherwise, similar protection would be expected from both the virulent Nine Mile I (NMI) and NMII (avirulent) vaccines, which is not observed. Moreover, the fact that the formalin-inactivated vaccine is effective without an adjuvant indicates that Cb expresses sufficiently immunostimulatory PAMPs, despite the minimal innate immune activation observed during live infection. This implies the existence of an as-yet-unidentified pathway involved in the recognition of the Cb O-antigen that confers intrinsic adjuvant activity [78,79].

### 5.2. Potential Coxiella burnetii Immunization Methods

Our group and others have observed that NMII requires a potent adjuvant to achieve protection, whereas NMI is fully protective without an adjuvant [80]. The PAMPs expressed by Cb include LPS, lipoproteins, peptidoglycan, DNA, RNA, and other unknown PAMPs. However, there is very little information on how these PAMPs specifically interact with their potential cognate PPRs other than for LPS. In addition, in vitro infection does not cause a large pro-inflammatory response, making it difficult to assess the big picture. Although Cb expresses many other PAMPs, limiting their access to the cytoplasm would prevent activation. The slow division rate of Cb, for example, would limit the release of peptidoglycan fragments which can be recognized by NOD1 and NOD2, and its vacuolar location further limits the access of these cytoplasmic receptors to byproducts. Therefore, although Cb expresses peptidoglycan, lipoproteins, DNA, and RNA, direct signaling through PPRs using purified species remains to be determined. In vitro infection suggests that none of these pathways are excessively stimulated because Cb can replicate in primary macrophages for more than a week [1]. Paradoxically, Q-Vax induces a prolonged antiviral state following immunization, suggesting robust type I interferon induction and implicating potent PAMPs capable of activating pathways such as RIG-I, cGAS–STING, or other interferon-associated sensors [79,80]. Thus, although the specific PAMPs responsible remain unidentified, their presence is strongly supported by vaccine efficacy.

### 5.3. Coxiella burnetii Virulence, Pathotypes, and Host Innate Immunity

The majority of BSL-3-level Cb work utilizes the virulent strain NMI. This strain possesses the unmodified and fully virulent LPS molecule as well as an extensive cohort of protein effectors. There are differences between the host immune response in guinea pigs to NMI and NMII (avirulent strain) as well as among any of the other numerous Cb pathotypes [81]. Specifically, guinea pigs infected intraperitoneally with NMI, Ohio, and NM Crazy strains all exhibited fever, while animals infected with NMII, Dugway, or Henzerling 331 strains did not. This same pattern held true for loss of body weight, where the same strains that induced fever also saw decreases in animal mass early in the course of infection. Another study by Kumaresan et al. pointed out that NMI intraperitoneal infection induced more neutrophil infiltrate in the lungs, spleen, and liver than was observed during NMII infection [82]. These studies highlight multiple experimental aspects that affect host response when studying Cb. NMI consistently produces a more intense host physiological and molecular response to infection, which brings into question the translatability of so many studies conducted using NMII as the main model. Additionally, many other pathotypes exist across the spectrum of intensity of host response, and the genetic differences between these pathotypes explaining their differing levels of virulence have yet to be fully explored. Finally, many studies employ peritoneal infection methods as opposed to aerosol, which further convolutes the questions surrounding the translatability of Cb animal infection models to human disease. Regardless of these differences, there have been some analyses that begin to point the field in the right direction to understanding the complex interplay between Cb virulence and the host response. Larson et al. posit that certain pathotypes possess partial or truncated sequences for key effectors that contribute to Cb pathogenesis and virulence, as supported by a genomic analysis of four key Cb strains [5]. We hypothesize that future investigations into these genomic differences will draw connections between more virulent Cb strains and conserved effectors.

### 5.4. Cell Culture Models for Coxiella burnetii Infection

An additional variable influencing interpretation of Cb-induced innate immune responses is the choice of the cellular model for in vitro infection studies. Although immortalized cell lines are experimentally tractable and sustainable, they often exhibit attenuated innate immune responses or require high multiplicities of infection to establish sustainable infection [83]. Previous work has shown that Cb prefers to infect macrophages, raising questions about the translatability of findings derived from immortalized cell lines or non-macrophage cell lines [84]. Even among primary macrophage models, the innate immune response varies substantially. BMDMs are widely used due to their accessibility and scalability; however, although Cb readily infects BMDMs, infection often elicits a more robust innate immune response than is characteristic of the bacterium’s native niche—alveolar macrophages [85]. BMDMs are typically skewed toward an M1-like phenotype (although this skew can be influenced with anti-inflammatory cytokine treatment) [86], whereas alveolar macrophages are more M2-like [87]. This difference in basal immune priming may influence the apparent importance of specific effectors discussed below during infection. Alternatively, BMDMs are invaluable models for demonstrating Cb effectors’ ability to inhibit innate immune processes that might otherwise remain silent in an M2-like macrophage, particularly in the context of further immune suppression during infection. While multiple cellular infection models are available for the study of Cb, careful consideration of the immune status and biological relevance of each model is essential when interpreting effector function and extrapolating conclusions to human disease.

### 5.5. Animal Models for Coxiella burnetii Infection

The study of Cb in vivo employs a variety of mammalian models, mostly mice and guinea pigs for small mammals and the occasional non-human primate model. Observations following infection differ across these models and merits a short discussion in order to place the experimental results in context when comparing studies using different animals. Neither mice nor guinea pigs develop pneumonia when infected via intraperitoneal injection, whereas they both experience marked pneumonia pathology in an aerosol infection [88,89,90]. Additionally, guinea pigs are more susceptible to Cb infection than mice, producing a high fever and granulomas in the bone, spleen, and liver (intraperitoneal infection) and more severe lung pathology (intratracheal) [91,92]. Non-human primates challenged with a Cb aerosol model recapitulate pneumonia lesions that are also observed in humans and are an ideal model for future Cb vaccine trials [92].

## 6. *Coxiella burnetii* Effectors That Inhibit the Innate Immune System

Cb-T4BSS translocates a highly diverse repertoire of effectors, most of which are unique to the *Coxiella* genus and lack homology to proteins in other organisms. To date, 150 T4BSS substrates have been identified, representing over 6% of the predicted protein-coding open reading frames in the Cb genome. Unlike *L. pneumophila*, which displays extensive functional redundancy among its effectors, Cb likely exhibits limited functional redundancy due to its smaller genome size and narrower host range. Multiple studies have identified Cb effectors that specifically target and inhibit the key innate immune pathways described above.

Although the precise functions and molecular mechanisms of many effectors remain incompletely understood, current evidence suggests that Cb is highly adapted to modulate nearly every major source of innate immune signaling encountered during a successful intracellular life. The following section summarizes recent advances in our understanding of T4BSS effector proteins that influence innate immune responses (Table 2).

### 6.1. CinF (CBU0513)

CinF is a Cb-T4BSS effector that inhibits NF-κB signaling. Its ~42 kDa protein that shares limited sequence similarity with ST0318 from *Sulfolobus tokodaii* and TnFBPAP from *Thermotoga neutrophilus*. Structural predictions suggest partial similarity to fructose-1,6-bisphosphatase-like proteins; however, CinF lacks detectable phosphatase activity toward most canonical substrates. The translocation of CinF from the CCV into the host cytoplasm has been demonstrated using a β-lactamase reporter assay in *L. pneumophila* [93].

Functional assays in PMA- or TNFα-stimulated HEK293T cells showed that CinF suppresses NF-κB activation, resulting in the accumulation of IκBα and impaired nuclear translocation of the p65 (RelA) subunit, indicating the inhibition of the canonical NF-κB pathway. Site-directed mutagenesis identified Tyr362 as critical for CinF-mediated sequestration of p65. Although CinF exhibited dephosphorylation activity toward phosphorylated IκBβ in vitro, a direct physical interaction between these proteins could not be demonstrated. Expression of CinF reduced p65 nuclear translocation by approximately 80%, and the knockdown of CinF significantly impaired Cb replication in HeLa cells [93]. While these findings strongly link CinF to NF-κB inhibition, definitive intracellular interaction partners have not yet been identified, a limitation common to many Cb effectors.

### 6.2. NopA (CBU1217)

NopA inhibits NF-κB signaling through an indirect mechanism involving the disruption of nucleocytoplasmic transport. NopA contains four RCC repeats within its C-terminal domain, a motif commonly associated with protein–protein interactions. NopA was also shown to interact with Ran-GTPase that regulates nuclear import/export by cycling between a GDP-bound cytosolic form and a GTP-bound nuclear form [64].

Although NopA lacks a canonical nuclear localization sequence, it preferentially binds GDP-bound Ran, thereby disrupting the Ran gradient required for efficient nuclear import of transcription factor p65. This interaction prevents the nuclear accumulation of p65 following stimulation, resulting in the attenuation of NF-κB-dependent transcription. While the precise subcellular localization of NopA remains unclear, these findings support a model in which Cb indirectly suppresses NF-κB signaling by interfering with Ran-dependent nuclear transport processes [64].

Importantly, the effects of NopA on nuclear import extend beyond NF-κB signaling. The disruption of Ran-dependent transport by NopA also impaired the nuclear translocation of IRF3 in response to Sendai virus infection in cells ectopically expressing NopA. These observations indicate that NopA broadly disrupts the nuclear import of immune TFs and suggest that NopA contributes to the inhibition of type I interferon responses during Cb infection by preventing IRF3 nuclear translocation [48,64].

### 6.3. EmcB (CBU2013)

EmcA and EmcB are required for (Cb)-mediated inhibition of RIG-I-like receptor (RLR) signaling. These effectors were identified through a transposon mutant screen for defects in suppressing poly(I:C)-induced activation of an interferon-stimulated response element (ISRE) luciferase reporter. Both EmcA and EmcB mutants impaired RIG-I suppression, and their complementation restored this phenotype, confirming their roles in RLR inhibition [48].

EmcA mutant displayed N an intracellular replication defect, EmcB mutant did not affect replication in macrophage-like cells. Importantly, impaired RIG-I inhibition could not be attributed solely to reduced replication, as other replication-deficient mutants retained the ability to suppress RLR signaling. Infection with either mutant resulted in elevated IFNB1 expression relative to wild-type infection, while increased IL-6 expression was observed specifically in the absence of EmcA, indicating overlapping but distinct immunomodulatory functions [48].

Mechanistically, EmcA and EmcB act through different modes of action. EmcA was required for RLR inhibition during infection but was not sufficient when ectopically expressed, suggesting a context-dependent role that may involve synergistic action with other effectors. In contrast, EmcB alone was sufficient to abolish RLR signaling by functioning as a deubiquitinase that removes K63-linked ubiquitin chains from RIG-I, a modification required for MAVS activation and downstream type I interferon production. EmcB also inhibited MDA5 signaling, indicating broader suppression of MAVS-dependent RLR pathways [48].

Together, these findings support a cooperative model in which EmcA functions as an infection-dependent regulator, while EmcB directly suppresses RLR signaling through deubiquitination, highlighting the layered strategies used by Cb to silence type I interferon responses.

### 6.4. IcaA (CBU1823)

IcaA (Inhibition of caspase activation) effector inhibits inflammasome activation by targeting the non-canonical inflammasome pathway. Infection of BMDMs with Cb resulted in the absence of caspase-1 cleavage and IL-1β secretion, indicating suppression of inflammasome activation. Because caspase-1 was not directly inhibited, upstream signaling events were examined. Using BMDMs deficient in NLRC4, NLRP3, or caspase-11, IcaA was shown to inhibit caspase-11-dependent signaling, thereby preventing the activation of the NLRP3 inflammasome. Consistent with caspase-11 inhibition, prior infection with Cb dampened subsequent inflammasome activation induced by *E. coli* LPS or cholera toxin B, which are established activators of the non-canonical inflammasome pathway [69].

As Cb infection alone does not robustly activate inflammasomes, heterologous infection models using *L. pneumophila* expressing IcaA were employed and demonstrated reduced inflammasome activation, further supporting the inhibitory role of IcaA. IcaA lacks recognizable protein domains and shares no significant homology with known bacterial or eukaryotic proteins, providing limited insight into its molecular mechanism [69].

The relevance of IcaA-mediated inflammasome inhibition in human macrophages remains to be fully defined. While IcaA inhibits caspase-11 activation in primary mouse AMs, recent studies have shown that NMII-Cb can induce IL-1β production in primary human AMs. Direct comparison of IcaA functions in human macrophages will be important for a comprehensive understanding of its role in host responses during Q fever.

### 6.5. CBU1314

CBU1314 is a nuclear-localized T4BSS effector that broadly suppresses host innate immune gene expression by interacting with the polymerase-associated factor 1 complex (PAF1C), a central transcriptional regulator of inflammatory pathways including MAPK, NF-κB signaling and ISGs [94]. Fischer et al. demonstrated that depletion of PAF1 phenocopied the effects of CBU1314 expression, resulting in suppression of NF-κB and interferon-dependent gene expression. Consistent with this, CBU1314 inhibited the transcription of key innate immune genes such as IRG1, TNF, IL6, and IL12B. Confocal microscopy further showed the localization of CBU1314 with chromatin in the nucleus, supporting its role as a transcriptional regulator [94].

In effector screening assays, CBU1314 exhibited one of the strongest inhibitory effects on NF-κB signaling in HEK293T reporter cells. Additional studies demonstrated that PAF1 is indispensable for restricting intracellular Cb replication, thereby identifying the PAF1 complex (PAF1C) as a key component of cell-intrinsic antibacterial defense. Notably, recent work has also implicated PAF1C in ISRE-dependent transcription, further linking CBU1314 activity to the suppression of type I interferon responses [94].

Collectively, these findings indicate that CBU1314 functions as a broadly acting suppressor of innate immune signaling by antagonizing PAF1-dependent transcriptional programs. The targeting of PAF1C by Cb highlights a central node through which multiple inflammatory and interferon pathways are simultaneously attenuated during infection.

**Figure 2:**
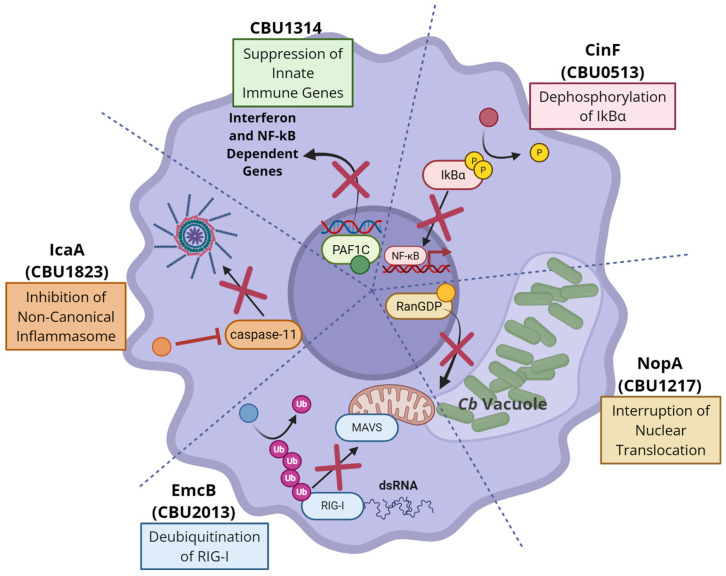
Innate immune inhibition mechanisms by selected *Coxiella burnetii* effectors. The five effectors discussed in detail in this review all play distinct roles in the suppression of host immunity. CBU1314 suppresses inhibition of a wide array of innate immune genes via competitive binding to the PAF1 complex. CinF is a dephosphorylase that inhibits propagation of signaling cascades in the NF-κB pathway. NopA inhibits nuclear translocation of innate immune transcription factors via binding to RanGDP. EmcB is a deubiquitinase that destabilizes RIG-I, a sensor of cytosolic dsDNA. IcaA inhibits caspase-11, a key enzyme in the non-canonical inflammasome cascade. Taken together, these 5 effectors downregulate vital host immune responses that would otherwise preclude Cb intercellular survival and replication.

As indicated earlier in this review, Cb possesses other effectors that work to inhibit cell death and therefore prolong the replication of the bacterium within macrophages. Three effectors, AnkG, CaeA, and CaeB, are hypothesized to inhibit apoptosis through a variety of mechanisms [95,96,97]. While a detailed explanation of their intracellular activity is outside the scope of this review, it is worth noting that such effectors exist, aiding in the maintenance of a calm and obliging cellular environment ideal for Cb survival. The five key effectors discussed above are summarized in Figure 2, painting a picture of the expansive, multi-pronged approach Cb takes to control the host innate immune response.

## 7. Conclusions and Future Perspectives

Over the past 15 years, the field of Cb research has made substantial progress in elucidating the functions of T4BSS effectors. As observed in the group of effectors described above, there seems to be a particular emphasis in Cb inhibiting the NF-κB and IFN signaling pathways due to their involvement in innate immune inflammation. Because it is likely that these particular inhibitions are critical to Cb survival, we would expect some redundant effectors to be characterized in the future, despite the more limited genomic capacity of the bacterium. Additionally, it is entirely possible that many of these effectors regulate each other or work in concert to optimally suppress host immune responses.

Despite encouraging advances in our comprehension of host-effector interactions, significant gaps remain. Beyond the many effectors that are still uncharacterized, the precise molecular mechanisms, context-dependent functions, and potential secondary or tertiary roles of several well-studied effectors are incompletely understood. Specifically, the investigations of many effector functions were conducted using in vitro infection models only and a lack the connection to animal infection models. Although many Cb effectors converge on host pathways targeted by other bacterial pathogens, they rarely share primary sequence homology with known effectors, complicating functional prediction and comparative analysis. Even state-of-the-art structural prediction platforms frequently generate low-confidence models for Cb proteins, highlighting current technical limitations in the field.

These challenges notwithstanding, advances in genetic manipulation, heterologous expression, and imaging approaches have substantially improved our ability to interrogate effector function in relevant in vitro systems. These approaches reveal how Cb uniquely exploits a lysosome-like compartment to support metabolic activation, differentiation, and effector delivery while maintaining remarkably weak innate immune activation. As these approaches continue to mature, additional effectors that modulate key nodes of innate immune signaling are likely to be identified, further expanding our understanding of the strategies employed by Cb to evade host immune surveillance.

## Figures and Tables

**Table 1:** Summary of discussed innate immune pathways responding to *Coxiella burnetii* infection.

Pathway	Stimulated by	Signals to
Toll-Like Receptors	LPS, zymosan, DNA, peptidoglycan, variety of Cb PAMPs	MyD88 or TRIF, then to Nuclear TFs
NLRs	TLRs engagement or PAMPs	Inflammasome
RIG-I-Like Receptors	Non-self RNA	MAVS
cGAS-STING	Cytoplasmic DNA	TANK1, Interferon
Type I/II Interferon	IFN-α/β/γ	ISRE/GAS in nucleus
NF-κB	TLRs	TNF-α, IL-1α/β, IL-2, IL-6, IL-17

**Table 2:** *Coxiella burnetii* effectors involved in innate immune inhibition.

Effector Name	Localization	Inhibited Pathway
CinF (CBU0513)	Cytoplasmic	NF-κB^80^
NopA (CBU1217)	Nuclear	Type I IFN/NF-κB^61^
EmcB (CBU2013)	Cytoplasmic	RIG-I^44^
IcaA (CBU1823)	Cytoplasmic	Inflammasome^81^
CBU1314	Nuclear	IFN/NF-κB^82^

## Data Availability

No new data were created or analyzed in this study.

## Abbreviations

The following abbreviations are used in this manuscript:

Cb	*Coxiella burnetii*
T4BSS	Type IVB secretion system
CCV	*Coxiella*-containing vacuole
PAMP	Pathogen-associated molecular pattern
LPS	Lipopolysaccharide
PRR	Pattern recognition receptor
SCV	Small-cell variant
LCV	Large-cell variant
BMDM	Bone marrow-derived macrophages
PTI	PAMP-triggered immunity
ETI	Effector-triggered immunity
TLR	Toll-like receptor
CLR	C-type lectin receptor
RLR	Retinoic acid-inducible gene I-like receptor
NLR	Nucleotide-binding domain, leucine-rich repeat-containing receptor
ZBP1	Z-DNA-binding protein 1
HAMP	Homeostasis-altering molecular pattern
DAMP	Damage-associated molecular pattern
NF-κB	Nuclear factor-κB
IRF3	Interferon regulatory factor 3
AP-1	Activator protein-1
CARD	Caspase activation and recruitment domain
GSDMD	Gasdermin D
NAIP	Neuronal apoptosis inhibitory protein
MAVS	Mitochondrial antiviral signaling protein
OMV	Outer membrane vesicle
TBK1	TANK-binding kinase 1
JAK-STAT	Janus kinase-signal transducer and activator of transcription
ISG	Interferon stimulated gene
iNOS	Inducible nitric oxide synthase
